# Associations between homocysteine and B vitamins and stroke: a cross-sectional study

**DOI:** 10.3389/fneur.2023.1184141

**Published:** 2023-06-29

**Authors:** Panpan Zhang, Xia Xie, Yurong Zhang

**Affiliations:** Department of Neurology, The First Affiliated Hospital of Xi'an Jiaotong University, Xi'an, China

**Keywords:** stroke, homocysteine, vitamin B6, folic acid, vitamin B12

## Abstract

**Objective:**

Homocysteine (Hcy) is a predictor for stroke. B vitamins are required for the metabolism of Hcy. We designed a study to investigate the associations of plasma Hcy and B vitamins with the prevalence of stroke in adults.

**Methods:**

A total of 8,371 adults were included in the National Health and Examination Survey (NHANES) between 2003–2006 in the United States. Multivariate regression analysis and smooth curve fitting were conducted to evaluate the associations of stroke prevalence with Hcy, folate, vitamin B6, and B12. A segmented regression model was used to analyze the threshold effects. Sample weights were calculated to ensure the results’ generalizability.

**Results:**

The mean age of all participants was 46.43 years (51.8% women), and the prevalence of stroke was 2.72%. A nonlinear and positive association was found between plasma Hcy levels and the prevalence of stroke. Furthermore, L-shaped associations were found between plasma vitamin B6 and folate levels and stroke, with the turning point at 65.2 nmol/L for vitamin B6 and 26 nmol/L for folate, respectively. Vitamin B12 revealed a U-shaped relationship with stroke, with the turning points at 492.98 pmol/L for vitamin B12.

**Conclusion:**

Non-linear associations of plasma Hcy and B vitamins levels with stroke prevalence were found in American adults. These associations may have an implication that higher plasma Hcy levels should be reduced, and plasma vitamin B6, vitamin B12 and folate levels should be moderately improved in stroke prevention. Future studies are needed to verify the causality of these associations and elucidate the underlying mechanisms.

## Introduction

Stroke is a major cause of death worldwide (1), with approximately 6.1 million deaths attributable to stroke (2). In older adults, stroke significantly contributes to disability and cognitive impairment by reducing movement in more than half of all survivors (3). Stroke prevention is a significant global public health challenge, and researchers have sought to reduce the incidence of stroke by eliminating its risk factors. Elevated plasma homocysteine (Hcy) is one of the most easily modifiable risk factors for stroke and is related to deficiencies in folate and vitamins B6 and B12 (3, 4).

Plasma Hcy is a type of sulfur-containing amino acid that is categorized as a reactive vascular injury amino acid due to its damage to vascular endothelial cells and subsequent occurrence of atherosclerosis and cerebrovascular diseases (5). Although some controversy exists, hyperhomocysteinemia is currently recognized as a risk factor for stroke (3, 4, 6). A recent meta-analysis showed that patients with ischemic stroke have higher levels of Hcy than controls; however, the cutoff of plasma Hcy and stroke risk is inconsistent in different populations (7). Levels of B vitamins and different backgrounds (sex and race/ethnicity) may contribute to the relationship between Hcy and stroke risk (8).

The B vitamins (folate, vitamin B6, and vitamin B12) are closely related to the metabolism of Hcy. Therefore, insufficient levels of B vitamins can lead to elevated plasma Hcy levels, which is associated with increased cardiovascular disease risk (9, 10). Implementing the folic acid food fortification policy has reduced plasma total Hcy concentrations (from 10.1 to 9.4 μmol per liter) and improved the levels of folate in the U.S. (11). A study showed that the population’s folic acid levels have remained relatively stable for some time and that there was a three-fold increase in the decline in stroke-related mortality in the U.S. after folic acid fortification (12). Lowering Hcy through vitamin B supplementation is a relatively inexpensive and simple approach; however, the associations between B vitamins and stroke are still controversial (13, 14).

Previous studies present differential results on the associations of Hcy and B vitamins levels with stroke after the folic acid fortification policy (15–17). Although previous studies have been conducted using a variety of study designs, including prospective cohorts, most have been conducted in non-U.S. settings and had relatively small sample sizes. They did not further analyze whether there was a linear relationship of plasma levels of Hcy and B vitamins with stroke or assess the thresholds for stroke risk. Therefore, we aimed to examine the associations of plasma levels of Hcy and B vitamins with stroke prevalence in U.S. adults using nationally representative data from the National Health and Nutrition Examination Survey (NHANES) after introducing mandatory folic acid fortification of flour.

## Materials and methods

### Study population

The present study is a cross-sectional analysis using the data from the NHANES 2003–2006. This study included individuals who participated in the 2003–2006 NHANES survey cycles and were older than 20 years of age. This included the most recent data on Hcy, folate, vitamin B6, and B12 that were released for public use. With a total of 11,189 participants, we excluded those with missing data, including Hcy (*n* = 1,659), stroke (*n* = 537), folate (*n* = 6), vitamin B6 (*n* = 83), and vitamin B12 (*n* = 130), and participants with missing data on covariates of interest (*n* = 403). The final sample size in the analysis was 8,371. All participants provided written informed consent prior to the surveys. The NHANES survey was approved by the Research Ethics Review Board of the National Center for Health Statistics, and the procedures followed the principles of the Declaration of Helsinki. The NHANES data used in this study are publicly available; therefore, the study was deemed to not require ethical or administrative permission.

### Covariates

The selection of variates was based on clinical experience and previous literature (18, 19). Variables included age (years), sex (female vs. male), race (Hispanic American, non-Hispanic White, non-Hispanic Black, or other), marital status (married or living with a partner, widowed/separated/ divorced, or never married), annual household income (<$20,000, $20,000–49,999, $45,000–$74,999, ≥$75,000), smoking status (current, former, or never), education (less than high school, high school, some college, college graduate or above), and body mass index (BMI). Histories of hypertension, diabetes, and stroke were recorded.

BMI was calculated as the weight (kg)/height square (m^2^). Data on the history of stroke were obtained from the questionnaire and assessed using the question, “Have you ever been told by a doctor or other health professional that you had a stroke?” Hypertension was defined as: (1) average systolic blood pressure/average diastolic blood pressure ≥ 140/90 mmHg, (2) previous diagnosis by a doctor or health professional, or (3) currently taking antihypertensive medications. Diabetes was defined as follows: (1) based on participants’ self-reported diagnosis of diabetes, or (2) fasting HbA1c greater than 6.4%.

Plasma total Hcy levels were measured by a fluorescence polarization immunoassay (Abbott Laboratories). Serum folate and vitamin B12 concentrations were measured simultaneously by the National Center for Environmental Health at the Centers for Disease Control and Prevention using a radioprotein binding assay kit (Quantaphase II; Bio-Rad Laboratories), and serum vitamin B6 (pyridoxal-5′-phosphate, PLP) concentration was measured by the high-performance liquid chromatography method using fluorometric detection (20, 21).

### Statistical analysis

Descriptive data on participants’ characteristics were expressed as means and SEs (standard error) or medians and interquartile ranges (IQRs) for continuous variables, and numbers and weighted percentages for categorical variables. One-way analysis of variance and chi-square tests were used to compare continuous and categorical variables, respectively. The weight prevalence of stroke was evaluated by age groups (20–29.9, 30–39.9, 40–49.9, 50–59.9, 60–69.9, and ≥ 70 years) in all participants. Multivariate logistic regression analyses were used to estimate odds ratios (ORs) and 95% confidence intervals (CIs) for the associations of stroke with Hcy and B vitamins. Variables were entered in the multivariate logistic regression models if the value of p was ≤0.05 in the univariable analysis. Because age is a strong risk factor for stroke, all logistic regression analyses were first adjusted for age (Model 1). Model 2 was further adjusted for sex, race, education, BMI, marital status, and annual household income, and Model 3 was further adjusted for a medical history of hypertension and diabetes. Smooth curve fitting was used to evaluate the potential non-linear relationships of Hcy and B vitamins with stroke, and a segmented regression model was used to analyze the threshold effect. Stratification analysis was conducted on the relationships of Hcy with stroke by age, sex, annual household income, race, and medical history of hypertension and diabetes, to evaluate the possible modifiers. Data were weighted to ensure that they were representative of the U.S. population using complex survey sampling analysis methods. All data analyses were conducted using R software (version R-4.1.0; Cary, NC, USA) and EmpowerStats 4.1. Two-sided *p*-values of <0.05 were considered statistically significant.

## Results

### Characteristics of the study population

**Figure 1:**
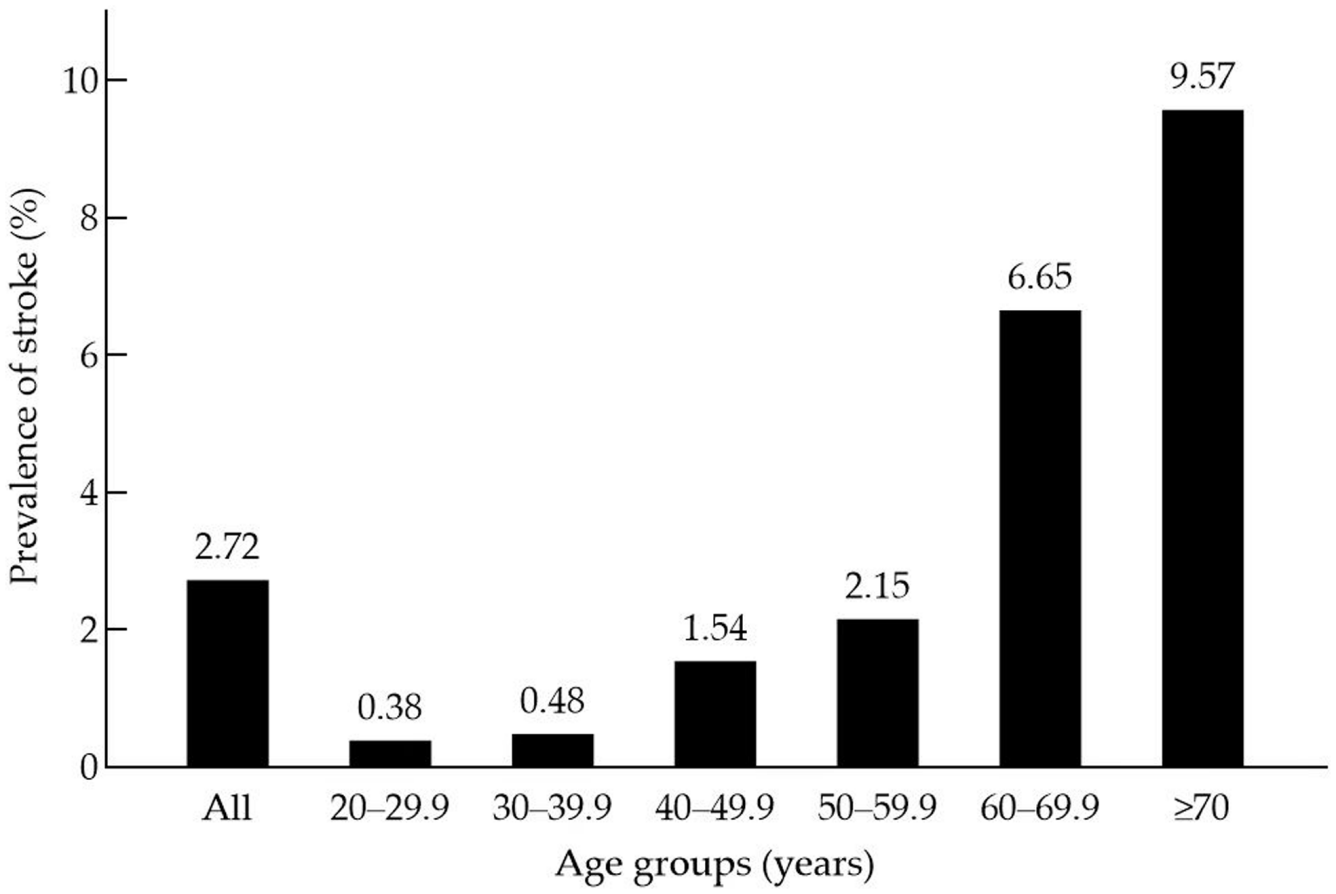
Prevalence (%) of stroke by age group, NHANES 2003–2006.

This study included 8,371 participants, representative of the total US population of 185,552,798 individuals. The mean age of the total group was 46.43 years, and 4,341 (51.80%) of the participants were women. Among the 8,371 participants, the weight prevalence of stroke was 2.72% (310). The weight prevalence of stroke increased with age: 0.38% in the age group 20–29.9 years, 0.48% in 30–39.9 years, 1.54% in 40–49.9 years, 2.15% in 50–59.9 years, 6.65% in 60–69.9 years, and 9.57% in ≥70 years, respectively (Figure 1).

Table 1 presents the characteristics of the participants in total and stratified by stroke status. Compared to those without stroke, participants with stroke were more likely to be older, female, non-Hispanic White, have a higher BMI, a lower annual household income, and were less likely to be educated. Additionally, they were more likely to have a history of hypertension and diabetes. There were no differences in the distribution of smoking. Participants with stroke were more likely to have higher levels of Hcy and folic acid and lower levels of vitamin B6. There was no difference in the distribution of vitamin B12 concentration.

**Table 1:** Characteristics of study participants by stroke, NHANES 2003–2006.

Characteristic	Total (*n* = 8,371)	No stroke (*n* = 8,061)	Stroke (*n* = 310)	*p*-value
Age, in years^a^	46.43 (0.46)	45.93 (0.45)	64.48 (1.28)	<0.001
Female, *n* (%)	4,341 (51.80)	4,188 (51.55)	153 (60.68)	0.011
Race, *n* (%)				0.006
Hispanic American	1,664 (7.37)	1,620 (7.79)	44 (4.23)	
Non-Hispanic White	4,372 (71.61)	4,183 (72.54)	189 (77.10)	
Non-Hispanic Black	1,734 (10.71)	1,672 (11.03)	62 (11.59)	
Multiracial or other	601 (8.52)	586 (8.70)	15 (7.07)	
Education Status, %				< 0.001
Less than high school	2,335 (16.97)	2,208 (16.99)	127 (29.15)	
High school	2,040 (25.49)	1,972 (25.76)	68 (25.40)	
Some college	2,356 (31.01)	2,283 (31.77)	73 (25.62)	
College graduate or above	1,640 (24.74)	1,598 (25.49)	42 (19.84)	
Marital status, *n* (%)				<0.001
Married or living with partner	5,227 (64.08)	5,058 (65.87)	169 (55.36)	
Widowed/separated/divorced	1,828 (18.61)	1,703 (18.08)	125 (38.72)	
Never married	1,316 (15.53)	1,300 (16.05)	16 (5.91)	
Current smoking, *n* (%)	1,863 (24.63)	1,804 (24.64)	59 (24.20)	0.884
Body Mass Index (kg/m^2^)^a^	28.42 (0.15)	28.38 (0.15)	29.82 (0.49)	0.007
Income, *n* (%)				<0.001
<20,000	2,382 (19.39)	2,247 (19.33)	135 (36.15)	
20,000–44,999	2,804 (30.58)	2,691 (30.92)	113 (38.68)	
45,000–74,999	1,614 (22.74)	1,572 (23.33)	42 (16.53)	
75,000+	1,571 (25.51)	1,551 (26.42)	20 (8.63)	
Homocysteine (umol/L)^b^	8.05 (6.62,9.85)	8.02 (6.659, 9.73)	10.96 (8.38, 13.24)	<0.001
Folate (nmol/L)^b^	26.5 (19.0,37.4)	26.5 (19.0, 37.1)	30.4 (20.2, 45.1)	0.011
Vitamin B12 (pmol/L)^b^	339.5 (256.8, 450.9)	339.5 (258.3, 450.9)	327.7 (236.9, 479.0)	0.784
Vitamin B6 (nmol/L)^b^	47.3 (26.4, 83.2)	47.8 (26.7, 83.4)	38.0 (21.0, 74.9)	0.007
Medical history, *n* (%)				
Diabetes	1,176 (10.74)	1,066 (10.11)	110 (33.46)	<0.001
Hypertension	3,429 (36.63)	3,175 (36.78)	255 (80.69)	<0.001

^a^Mean (SE), ^b^Median (interquartile ranges).

### Association between Hcy and B vitamins and stroke

Table 2 shows the results of the multivariate logistic regression analysis. When Hcy was classified as a quintile categorical variable using the first quintile as a reference, the ORs (95% CI) of stroke after full adjustment were 1.00 (ref), 1.19 (0.51, 2.78), 1.16 (0.53, 2.57), 1.21 (0.56, 2.58), and 2.83 (1.15, 6.98) across increasing Hcy quintiles, respectively. Plasma vitamin B6 showed an L-shaped association with stroke prevalence, with the aORs (95% CI) being 1.00 (ref), 0.76 (0.49, 1.17), 0.75 (0.50, 1.12), 0.52 (0.28, 0.98), and 0.57 (0.36, 0.89) across increasing vitamin B6 quintiles, respectively. A likelihood of U-shaped association was found between vitamin B12 and stroke, with the aORs (95% CI) being 1.00 (ref), 0.65 (0.38, 1.12), 0.65 (0.36, 1.18), 0.58 (0.32, 1.01), and 0.84 (0.53, 1.34), respectively.

**Table 2:** Adjusted associations between homocysteine and B vitamins and stroke, NHANES 2003–2006.

Variables	Sample size (*n*)	Model 1 OR (95% CI)	*p*-value	Model 2 OR (95% CI)	*p*-value	Model 3 OR (95% CI)	*p*-value
Homocysteine Categorical^a^							
Quintile 1 (<6.22)	1,668	Reference		Reference		Reference	
Quintile 2 (6.22–7.50)	1,665	0.91 (0.36, 2.30)	0.727	1.24 (0.56, 2.79)	0.569	1.19 (0.51, 2.78)	0.658
Quintile 3 (7.50–8.80)	1,683	0.91 (0.45, 1.83)	0.943	1.20 (0.54, 2.64)	0.629	1.16 (0.53, 2.57)	0.685
Quintile 4 (8.80–10.89)	1,680	1.02 (0.54, 1.93)	0.685	1.34 (0.64, 2.78)	0.406	1.21 (0.56, 2.58)	0.598
Quintile 5 (≥10.89)	1,675	2.55 (1.26, 5.13)	0.012	3.32 (1.38, 7.97)	0.011	2.83 (1.15, 6.98)	0.028
Vitamin B12 Categorical^a^							
Quintile 1 (<239.11)	1,662	Reference		Reference		Reference	
Quintile 2 (239.11–308.48)	1,677	0.67 (0.41, 1.08)	0.097	0.67 (0.40, 1.12)	0.119	0.65 (0.38, 1.12)	0.109
Quintile 3 (308.48–383.02)	1,677	0.61 (0.35, 1.06)	0.076	0.64 (0.36, 1.14)	0.121	0.65 (0.36, 1.18)	0.143
Quintile 4 (383.02–498.89)	1,679	0.57 (0.33, 0.98)	0.044	0.60 (0.34, 1.04)	0.067	0.58 (0.32, 1.01)	0.055
Quintile 5 (≥498.89)	1,676	0.79 (0.52, 1.20)	0.254	0.85 (0.55, 1.32)	0.446	0.84 (0.53, 1.34)	0.431
Vitamin B6 Categorical^a^							
Quintile 1 (<21.1)	1,667	Reference		Reference		Reference	
Quintile 2 (21.1–35.8)	1,674	0.71 (0.47, 1.07)	0.094	0.75 (0.49, 1.14)	0.162	0.76 (0.49, 1.17)	0.190
Quintile 3 (35.8–55.2)	1,679	0.68 (0.47, 0.97)	0.037	0.75 (0.51, 1.11)	0.135	0.75 (0.50, 1.12)	0.145
Quintile 4 (55.2–91.4)	1,676	0.42 (0.24, 0.74)	0.004	0.49 (0.26, 0.92)	0.029	0.52 (0.28, 0.98)	0.045
Quintile 5 (≥91.4)	1,675	0.46 (0.30, 0.70)	<0.001	0.57 (0.36, 0.90)	0.020	0.57 (0.36, 0.89)	0.019
Folate Categorical^a^							
Quintile 1 (<17.4)	1,648	Reference		Reference		Reference	
Quintile 2 (17.4–23.3)	1,674	0.75 (0.40, 1.39)	0.343	0.79 (0.42, 1.48)	0.432	0.75 (0.39, 1.43)	0.347
Quintile 3 (23.3–29.9)	1,673	0.66 (0.36, 1.19)	0.156	0.71 (0.38, 1.33)	0.258	0.67 (0.36, 1.26)	0.193
Quintile 4 (29.9–40.8)	1,688	0.62 (0.33, 1.16)	0.128	0.70 (0.36, 1.36)	0.272	0.67 (0.34, 1.31)	0.215
Quintile 5 (≥40.8)	1,688	0.65 (0.36, 1.17)	0.144	0.75 (0.40, 1.42)	0.353	0.72 (0.38, 1.39)	0.297

^a^Unit: Homocysteine: umol/L; vitamin B6: nmol/L; vitamin B12: pmol/L; folate: nmol/L. Model 1: Age. Model 2: Model 1+ sex, education, race, marital status, body mass index, income. Model 3: Model 2 + history of hypertension, diabetes.

**Figure 2:**
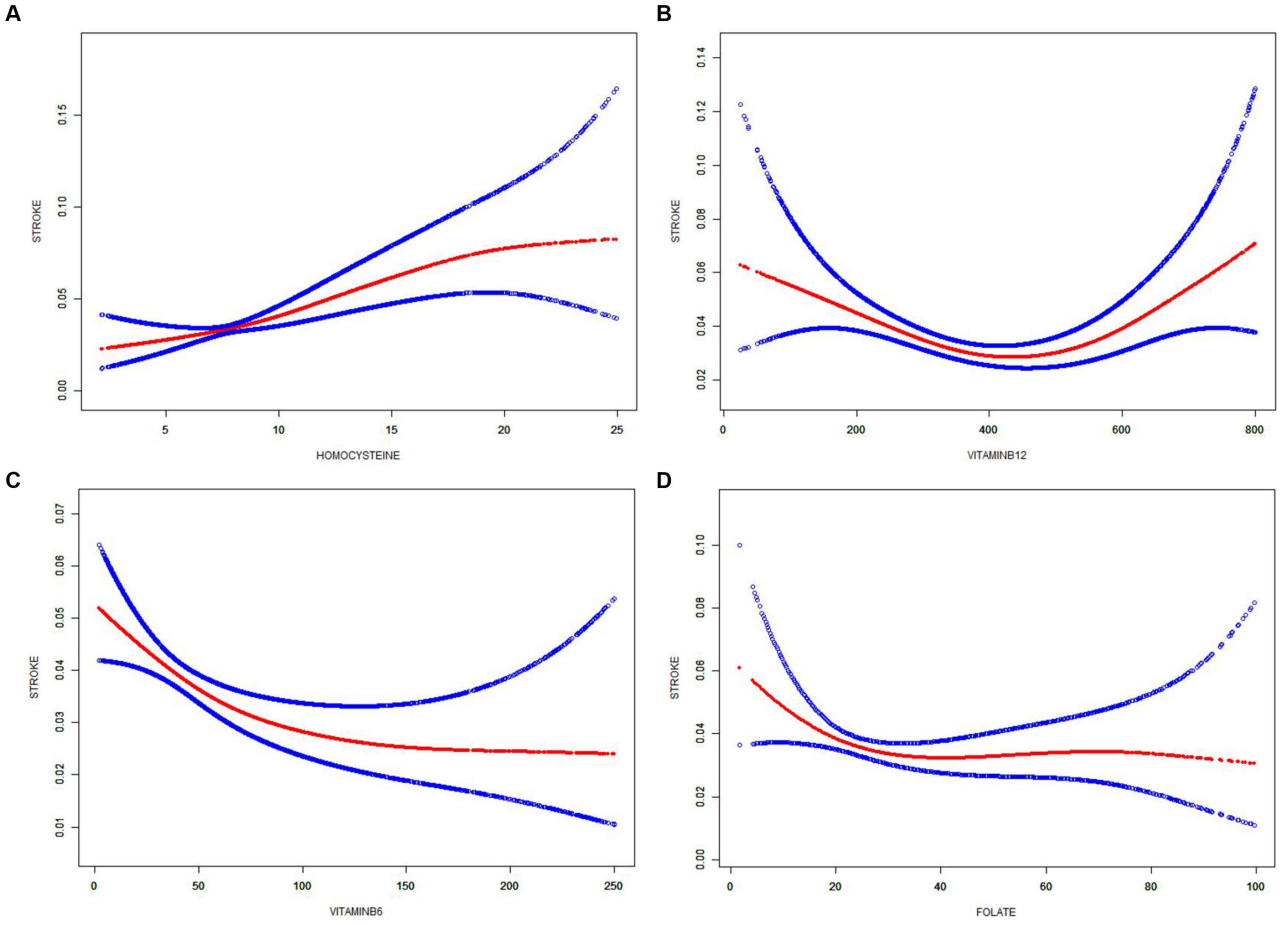
Smooth curve fitting shows the relationships of homocysteine and B vitamins with stroke. Smoothing curve of **(A)** Homocysteine, **(B)** Vitamin B6, **(C)** Vitamin B12, and **(D)** Folate Non-linear plots are displayed with red lines, and blue dotted lines are the 95% confidence intervals (CI). ORs (95% CI) were adjusted based on Model 3.

The associations of plasma Hcy and vitamins B with stroke prevalence using spline smoothing fittings can be seen in Figure 2. A positive relationship was found between Hcy and stroke prevalence (adjusted OR = 1.114, 95% CI: 1.063, 1.167); however, increased plasma Hcy level was no longer associated with a further increased stroke prevalence (adjusted OR = 0.996, 95% CI: 0.996, 1.027) in participants with a plasma Hcy concentration > 15.3 umol/L (logarithmic likelihood ratio test (LRT): *p* < 0.001) (Figure 2A; Table 3).

**Table 3:** Threshold effect analysis for the relationship between homocysteine and B vitamins and stroke.

Threshold effect analysis^a^	Adjusted OR (95% CI)	*p*-value
Homocysteine		
Model I		
One line slope	1.025 (1.010, 1.041)	0.001
Model II		
Turning point (K)	15.3	
<15.3 slope 1	1.114 (1.063, 1.167)	<0.001
>15.3 slope 2	0.996 (0.996, 1.027)	0.818
Slope 2-Slope 1	0.894 (0.840, 0.953)	<0.001
Predicted at 15.3	−1.706 (−1.919, −1.493)	
LRT test	<0.001^*^	
Vitamin B12		
Model I		
One line slope	0.9996 (0.9989, 1.0004)	0.325
Model II		
Turning point (K)	492.98	
<492.98 slope 1	0.998 (0.997, 0.999)	0.009
>492.98 slope 2	1.002 (1.000, 1.003)	0.046
Slope 2-Slope 1	1.003 (1.001, 1.006)	0.009
Predicted at 492.98	−3.650 (−3.897, −3.402)	
LRT test	0.011	
Vitamin B6		
Model I		
One line slope	0.998 (0.996, 1.000)	0.018
Model II		
Turning point (K)	65.2	
<65.2 slope 1	0.989 (0.983, 0.995)	<0.001
>65.2 slope 2	1.000 (0.998, 1.002)	0.866
Slope 2-Slope 1	1.011 (1.004, 1.019)	0.004
Predicted at 65.2	−3.518 (−3.725, −3.311)	
LRT test	0.004^*^	
Folate		
Model I		
One line slope	0.998 (0.994, 1.003)	0.473
Model II		
Turning point (K)	26	
<26 slope 1	0.970 (0.945, 0.995)	0.020
>26 slope 2	1.000 (0.997, 1.004)	0.914
Slope 2-Slope 1	1.0312 (1.004, 1.060)	0.025
Predicted at 26	−3.243 (−3.387, −3.099)	
LRT test	0.030^*^	

^a^Unit: Homocysteine: umol/L; vitamin B6: nmol/L; vitamin B12: pmol/L; folate: nmol/L. Adjustment was performed for age, sex, education, race, marital status, body mass index, income, history of hypertension, diabetes. Model I, linear analysis; Model II, non-linear analysis. LRT test, logarithmic likelihood ratio test. ^*^Indicates that Model II is significant different from Model I.

A U-shaped relationship between plasma vitamin B12 and stroke prevalence was found. The threshold effect analysis showed that there was an inverse association between vitamin B12 and stroke in participants with vitamin B12 < 492.98 pmol/L, and a positive association between vitamin B12 and stroke in those with vitamin B12 > 492.98 pmol/L (Figure 2B; Table 3).

An L-shaped relationship between vitamin B6 and stroke prevalence was observed (Figure 2C). In the threshold effect analysis, the prevalence of stroke significantly decreased as the plasma vitamin B6 concentration increased (per unit increment: adjusted OR = 0.989, 95% CI: 0.983, 0.995) in participants with a plasma vitamin B6 concentration < 65.2 nmol/L; however, increased plasma vitamin B6 was no longer associated with a decreased stroke prevalence (adjusted OR = 1.000, 95% CI: 0.998, 1.002) in participants with a plasma vitamin B6 concentration > 65.2 nmol/L (LRT test: *p* = 0.004). Similar results were found between plasma folate and stroke (Figure 2D). Stroke prevalence decreased by 3.04% per unit with increasing plasma folate levels up to the turning point (26 nmol/L) (adjusted OR = 0.970, 95% CI: 0.945, 0.995), and stroke prevalence was no longer decreased with folate levels when folate was >26 nmol/L (adjusted OR = 1.000, 95% CI: 0.997, 1.004) (LRT test: *p* = 0.030) (Table 3).

### Stratification analysis

**Figure 3:**
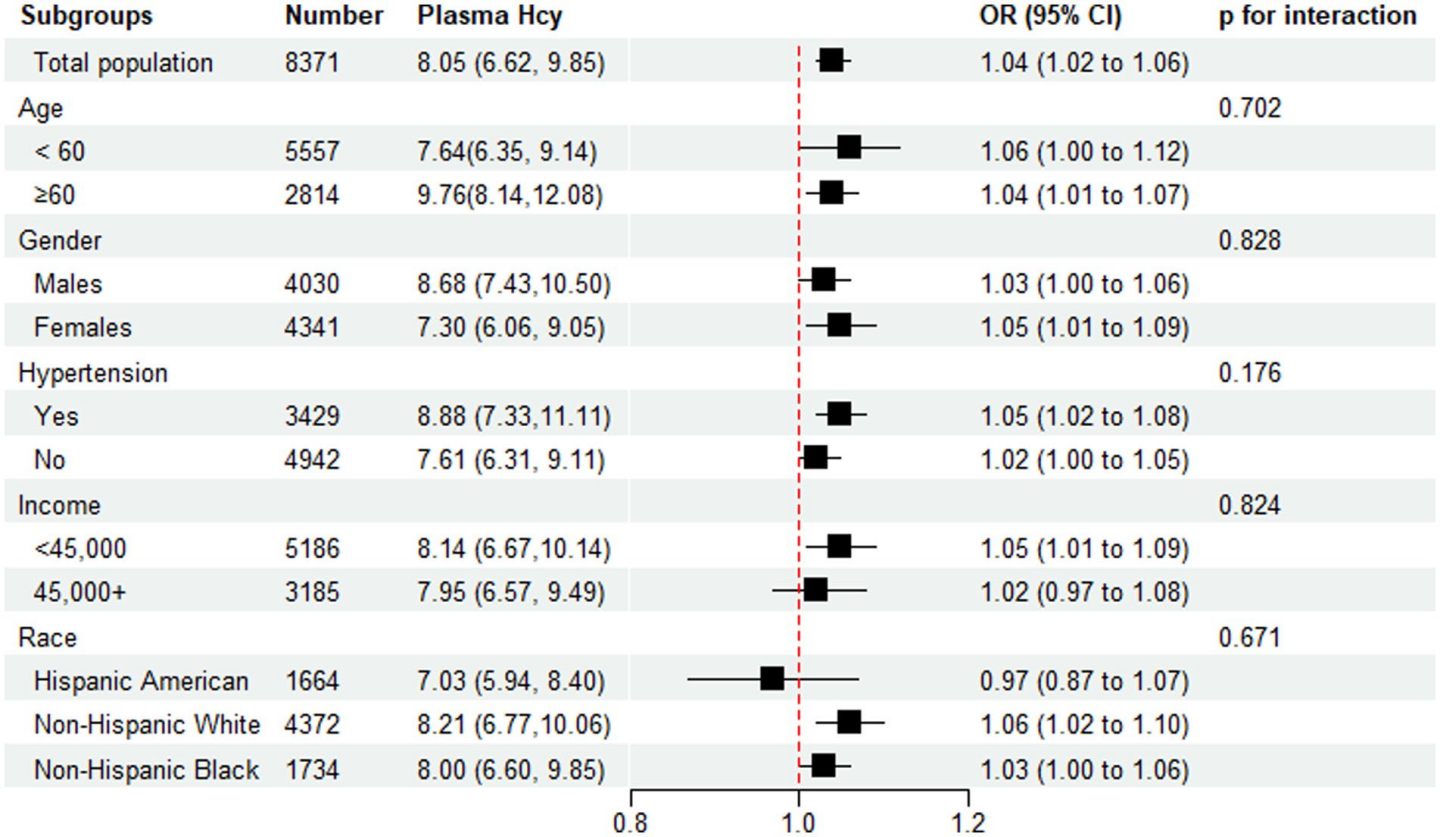
Subgroup analysis of the association of Homocysteine with stroke. Results were adjusted for all covariates except the corresponding stratification variable.

Subgroup analysis was performed by age, sex, race, income, and hypertension (Figure 3). Hcy was significantly associated with stroke in all subgroups. As shown in Figure 3 of the forest plot, no interactions were observed between plasma Hcy concentration and age (p-interaction=0.702), sex (p-interaction = 0.828), race (p-interaction = 0.671), income (p-interaction = 0.824), and hypertension (p-interaction = 0.176); therefore, none of these variables significantly modified the association between Hcy and stroke. Even though the Hcy–stroke association was slightly stronger in non-Hispanic White subjects (OR = 1.06, 95% CI: 1.02–1.10) and lower-income individuals (OR = 1.05, 95% CI: 1.01–1.09) (combined <20,000 and 20,000–44,999, since no difference was found between these two groups regarding the ORs), no interaction existed statistically (all p interactions >0.05).

## Discussion

In the current large cross-sectional study with a representative U.S. sample population, we found that plasma Hcy levels were nonlinearly and positively associated with stroke prevalence, and stroke prevalence was no longer increased when plasma Hcy concentration was >15.3 umol/L. Furthermore, L-shaped associations were identified between plasma vitamin B6 and folate and stroke prevalence, with the turning point of 65.2 nmol/L for vitamin B6 and 26 nmol/L for folate, respectively. Vitamin B12 revealed a U-shaped relationship with stroke prevalence, with the turning point at 492.98 pmol/L. These findings may provide clinical and nutritional implications for stroke prevention.

The positive association between Hcy and stroke has been confirmed in previous studies (22–25). A recent review found a 43% increased risk of ischemic stroke with a 5 μmol/L increase in Hcy levels (23). An elevated Hcy level was reported to positively associate with high risk of intracerebral hemorrhage (26). A meta-analysis of prospective cohort studies found a linear association between Hcy level and total stroke (Pnonlinearity = 0.660). For each 1-μmol/L increase in Hcy, stroke risk increases 1.06 times (25). However, in a cohort study of U.S. male physicians, Hcy did not have a significant association with ischemic stroke (8). In the present study, we found a non-linear and positive relationship between plasma Hcy levels and total stroke in the folate-fortified population. The inconsistent results on the relationship between Hcy and stroke may be due to different backgrounds, because Hcy levels are affected not only by vitamin deficiency (such as folic acid, vitamin B6, and B12), but also by factors such as sex, diet, and genetic mutations (8). In addition, unlike in previous studies, we used the methods of smooth curve fitting, segmented regression model, and LRT test in the present study. The application of these statistical methods in the field of stroke is novel and can be used to find the exact association when there is a possible threshold effect.

Furthermore, we performed stratified analyses to determine the relationship between Hcy and stroke by sex, hypertension, ethnicity, and income. In contrast to previous studies (22, 24), this study demonstrated no interaction between plasma Hcy concentration and hypertension (p-interaction = 0.176), indicating that Hcy was significantly associated with stroke in individuals with and without hypertensive. Hypertension was not a modifier of the relationship of Hcy with stroke. Similar to our results, a Chinese study found no interaction between plasma total Hcy concentration and blood pressure (p-interaction = 0.889) (27), and a Japanese study showed no significant difference in the association between Hcy and stroke after stratification by blood pressure levels (18), indicating that Hcy was associated with stroke independently of hypertension. Hcy is currently considered a useful marker for inflammation and may aggravate the process of atherosclerosis and elevate the risk of stroke by the pathogenesis such as inflammatory reaction, oxidative stress, and coagulation dysfunction (28).

We found that the Hcy level in men was slightly higher than in women; however, sex differences were not observed in the association between Hcy and stroke. Similar results were found in a study conducted by NHANES III before folic acid fortification (19). A review based on 12 epidemiological studies suggested that hyperhomocysteinemia should be considered a possible risk factor for vascular disease in both men and women, before and after menopause. A study in the review reported that elevated Hcy was a stronger predictor for vascular disease (including ischemic stroke) in women than in men, and the stronger correlation among women may explained by the study design, such as age at inclusion (women usually suffer from vascular diseases later in life than do men) (29).

Consistent with the findings of a case–control study of stroke in young women (30), the present study found that Hcy was independent of poverty. However, poverty was strongly associated with stroke, and the intensity of the association between Hcy and stroke remained significant but decreased following adjustment for income. This may be because low-income individuals are more likely to be exposed to traditional high-risk behaviors, such as smoking, malnutrition, lower vitamin intake, lack of preventive medicine, and lack of treatment for high blood pressure (31).

Our study showed an L-shaped association between folate and stroke in the U.S. after folic acid fortification. The protective effect of folic acid on stroke has been debated (13, 14). Recent meta-analyses showed that folic acid has a significant protective effect on stroke (32). In practice, reducing plasma Hcy by folic acid supplementation may be affected by the status of folic acid in the population, and the amount of Hcy reduction in areas without folic acid fortification is significantly higher than that in areas with folic acid fortification (33). The present study found that stroke prevalence decreased with increasing plasma folate levels up to 26 nmol/L, and stroke prevalence was not further decreased when folate was >26 nmol/L, suggesting that very high folate levels did not appear to have an additional benefit on decreasing stroke prevalence. Similar to our study, Shirodaria et al. found that folic acid supplementation with a low dose (0.4 mg/day) was enough to improve vascular endothelial function, whereas increasing the dose to 5 mg/day had no additional benefit (34), indicating that high-dose folic acid treatment likely confers no further benefit in subjects already receiving folate supplementation.

This study also identified an L-shaped association between vitamin B6 and stroke prevalence, with a turning point of 65.2 nmol/L. A previous study conducted in a U.S. hospital population from 1999 to 2001 showed that individuals with lower B6 levels had an increased risk of stroke (35). Moreover, vitamin B6 was found to have a protective effect on the occurrence and prognosis of cardiovascular events among stroke patients. Conversely, excess vitamin B6, achieved with improper supplementation, has been proven to be related to toxic effects, including neurological and gastrointestinal disturbances (36).

The present study showed a U-shaped association between vitamin B12 and stroke prevalence. Vitamin B12 deficiency is a nutritional determinant of total Hcy and is associated with a poor stroke prognosis (37). However, the direct relationship between vitamin B12 and stroke has not been well studied (38). The Nurses’ Health Study found that a high intake of both vitamins (B6 ≥ 35 mg/d and B12 ≥ 20 μg/d) was associated with an increased risk of hip fracture compared to a low intake of both vitamins (B6 < 2 mg/d and B12 < 10 μg/d), suggesting that vitamin B supplementation should be used with caution because adverse effects can occur (39). The U.S. Preventive Services Task Force (USPSTF) concluded that the current evidence is insufficient to assess the balance of benefits and harms of the use of multivitamin supplements for the prevention of cardiovascular disease (I statement) (40). A dose–response meta-analysis based on 11 prospective studies showed increased intake of vitamin B-6 and folate is associated with a reduced risk of stroke and no significant association between dietary vitamin B12 intake and stroke risk, suggesting that increasing habitual vitamin B6 and folate intake may provide a small but beneficial effect with respect to stroke (41). The precise mechanism underlying the effect of B vitamins on stroke is unknown; however, the putative beneficial effect of B-vitamins in lowering Hcy on stroke may be attributed to reduced atherosclerosis and increased vascular-protective properties, meanwhile, excess B vitamins were related to toxic effects.

Despite the critical findings of our study, some limitations should be mentioned. Firstly, NHANES is designed as a cross-sectional study and is observational in nature; therefore, causality cannot be determined, and residual confounding cannot be completely ruled out. Future longitudinal investigations are required to determine the predictive value of Hcy and B vitamins for stroke risk. Secondly, the self-reporting of stroke was likely to introduce bias. However, the questionnaire administered in this study has been widely used to assess stroke in previous studies (42, 43). Thirdly, although explicit information regarding the subtypes of stroke in the NHANES database is lacking, our study sample is representative of a general adult population. Finally, the relationships explored in this study were based on the U.S. population (2003–2006 NHANES data), which is a country where folic acid fortification is practiced; therefore, caution must be taken when generalizing these findings in populations without folic acid fortification. Future studies should further explore the relationships of Hcy and B vitamins with stroke and its subtypes in different populations. Interventions and randomized controlled trials are needed to explore the relationships of Hcy lowering and B vitamins taking with stroke risk.

## Conclusion

In this large cross-sectional study, plasma Hcy level was positively and nonlinearly associated with stroke. Plasma vitamin B6 and folate had an L-shaped, and vitamin B12 had a U-shaped relationship with stroke, respectively. Higher plasma Hcy levels should be reduced and lower B vitamins levels should be moderately improved in the prevention of stroke. These results highlight the potential advantages of monitoring and evaluating Hcy and B vitamins status in the prevention of stroke. Prospective and mechanistic studies are necessary to elucidate the causality of these associations.

## Data availability statement

The datasets presented in this study can be found in online repositories. The names of the repository/repositories and accession number(s) can be found at: the official NHANES website (www.cdc.gov/nchs/nhanes/).

## Ethics statement

The NHANES survey was approved by the Research Ethics Review Board of the National Center for Health Statistics (NCHS) and the procedures followed the principles of the Declaration of Helsinki. Written informed consent for participation was not required for this study in accordance with the national legislation and the institutional requirements.

## Author contributions

YZ contributed to the conception and design of the study. PZ contributed to drafting the article. XX contributed to revising the article critically. All the authors have read and approved the manuscript.

## Funding

This research was funded by the Clinical Research Award of the First Affiliated Hospital of Xi’an Jiaotong University (No.XJTU1AF2018CRF-024) and by the horizontal subject of the First Affiliated Hospital of Xi’an Jiaotong University (Grant No. HX201872).

## Conflict of interest

The authors declare that the research was conducted in the absence of any commercial or financial relationships that could be construed as a potential conflict of interest.

## Publisher’s note

All claims expressed in this article are solely those of the authors and do not necessarily represent those of their affiliated organizations, or those of the publisher, the editors and the reviewers. Any product that may be evaluated in this article, or claim that may be made by its manufacturer, is not guaranteed or endorsed by the publisher.
